# Global Expression Profiling in Epileptogenesis: Does It Add to the Confusion?

**DOI:** 10.1111/j.1750-3639.2008.00254.x

**Published:** 2010-01

**Authors:** Yi Yuen Wang, Paul Smith, Michael Murphy, Mark Cook

**Affiliations:** Centre for Clinical Neuroscience and Neurological Research, St Vincent's HospitalMelbourne, Australia

**Keywords:** epileptogenesis, gene expression, hippocampus, serial analysis of gene expression, temporal lobe epilepsy

## Abstract

Since the inception of global gene expression profiling platforms in the mid-1990s, there has been a significant increase in publications of differentially expressed genes in the process of epileptogenesis. In particular for mesial temporal lobe epilepsy, the presence of a latency period between the first manifestation of seizures to chronic epilepsy provides the opportunity for therapeutic interventions at the molecular biology level. Using global expression profiling techniques, approximately 2000 genes have been published demonstrating differential expression in mesial temporal epilepsy. The majority of these changes, however, are specific to laboratory or experimental conditions with only 53 genes demonstrating changes in more than two publications. To this end, we review the current status of gene expression profiling in epileptogenesis and suggest standard guidelines to be followed for greater accuracy and reproducibility of results.

## INTRODUCTION

The understanding of epilepsy as a complex interaction between numerous excitatory and inhibitory neuronal connections influenced at the molecular level rightly brings about focus to global changes in the entire transcriptome in the epileptogenic process. Annotating the individual genomic changes at specific time points delivers only a snapshot of a much larger process. The development of global gene expression profiling platforms has revolutionized molecular biological research, allowing vast amounts of information to be identified, catalogued and measured. Genomic changes are seen in concert giving the potential for a broader understanding of the disease process.

The late 1990s heralded the development of several important technological advances in molecular biological research. DNA microarray and serial analysis of gene expression (SAGE) were introduced allowing comparisons of global gene expression utilizing differing conditions in laboratory experimentation. This has allowed the relatively new field of comparative genomics to flourish (43, 54). With respect to the brain, patterns of gene expression have been reported as early as 1999 with comparative libraries generated between malignant glioma tissue and normal brain for SAGE, and between sleep–wake cycles for microarray (40, 55). From this point there has been an explosion of genomic information with steadily increasing reports generated during development and aging (38, 94, 106), malignant brain processes (37, 101), neurodegenerative disorders (41, 58), behavioral (12, 45) and anatomical studies (18). Studies into epileptogenesis have also expanded since the first global gene expression studies into epilepsy in 2001 (29, 42). For this reason we will review the published papers and discuss the usefulness of global transcriptome analyses with regards to epileptogenesis.

## EPILEPSY AND EPILEPTOGENESIS

Epilepsy is a disorder of the brain characterized by an enduring predisposition to generate epileptic seizures and by the neurobiological, cognitive, psychological and social consequences of this condition. An epileptic seizure is a transient occurrence of signs and/or symptoms caused by the abnormal excessive or synchronous neuronal activity in the brain (27). It includes seizures occurring in the absence of a recognized cause as well as events occurring in patients with antecedent stable (non-progressing) central nervous system insults (unprovoked seizures), but excludes seizures occurring in a close temporal relationship with an acute systemic, toxic or metabolic insult, which is expected to be the underlying cause (provoked seizures) (28).

**Figure 1:**
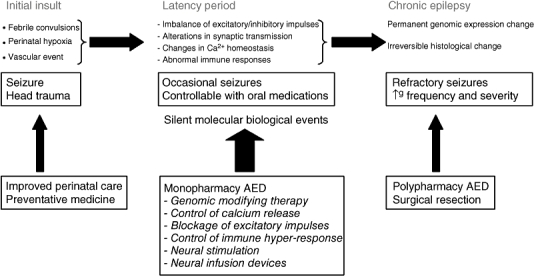
*Initial hit theory of epileptogenesis.* Abbreviations: AED = anti-epileptic drug.

Within the broad classification of epilepsy, mesial temporal lobe epilepsy (MTLE) is the most common location-related epilepsy. This may be lesional or non-lesional, with the great majority of non-lesional MTLE being associated pathologically with hippocampal sclerosis (HS). There remains much debate in the neurological world regarding HS as a cause or consequence of seizures. Pathological evidence has shown the presence of HS in brains of dementia patients without clinical epilepsy (19, 104). In addition, MTLE patients with dual pathology may have HS that is: (i) a non-specific result of the primary epileptogenic lesion and not in itself epileptogenic; (ii) secondary to the primary epileptogenic lesions but also epileptogenic; or (iii) a primary HS that coexists with another epileptogenic lesions (109). Proponents of the initiating hit theory of epilepsy argue that the progressive nature of MTLE demonstrates HS as a consequence of the seizure process. Typically a variable latent period between the initial precipitating incident and habitual unprovoked seizures is present, followed by a silent period between the first habitual unprovoked seizure and the onset of intractability. This strongly suggests that the pathology is progressive (5). Worsening HS creates a cycle whereby increased seizure severity and frequency lead to further neuropathological changes and hence worsening clinical seizures. Several theories for the cause of HS have been proposed, with the most commonly quoted being febrile convulsions. Other theories include vascular injuries and developmental abnormalities. It is this process of progression to seizures of worsening severity with increasing frequency that is termed epileptogenesis (Figure 1).

**Figure 2:**
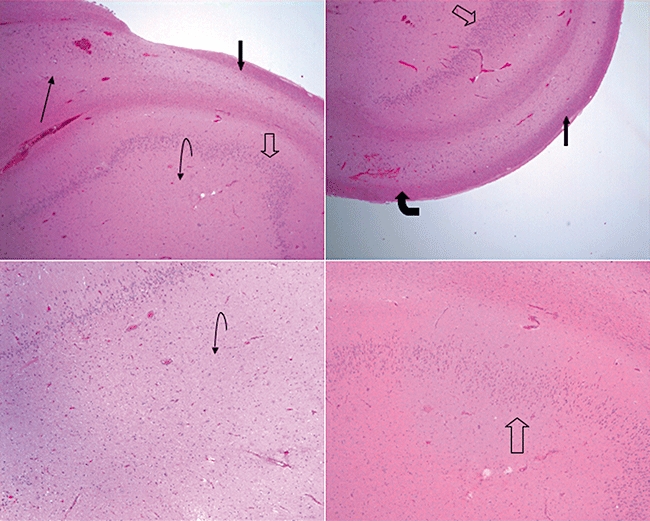
(L-top) Low power (2.5×) view demonstrating normal subiculum into CA1; (R-top) Low power (2.5×) view demonstrating CA1 sclerosis and sparing of CA2; (L-bottom) Low power (2.5×) view demonstrating neuronal loss in CA3/4; (R-bottom) Medium power (5×) view with dispersion of dentate gyrus. (arrow: subiculum, block arrow: CA1, curved block arrow: CA2, curved arrow: CA3/4, arrow outline: dentate gyrus).

HS was first described in 1825 by Bouchet and Cazauvieil (8). It is a pathological diagnosis although advances in neuroimaging through magnetic resonance imaging had allowed the use of radiology as a supporting or diagnostic tool. The pathological diagnosis consists of atrophy of the hippocampal formation with loss of neurones and reactive gliosis in CA1, CA4 and the dentate gyrus. In CA4, there is a loss of polymorphic neurons and pyramidal neurons. CA3 shows some neuronal loss, but CA1 always shows significant neuronal loss that may be very severe. There is a variable degree of gliosis seen in these subregions and occasional vessel sclerosis has been described. Loss of granule cells in the dentate gyrus is variable, as is gliosis. Granule cell distribution is often disrupted and various patterns including dual lamination or dispersion are described. Sprouting of the mossy fiber system in the dentate gyrus is apparent with Timm's stain with many believing that this sprouting leads to aberrant reinnervation resulting in the hyperirritable lesion that constitutes the epileptogenic hippocampus. Within all these changes, CA2 is relatively spared (68) (Figure 2).

Treatment of non-lesional MTLE continues to be a challenge for the epileptologist. Monodrug therapy is initially implemented however this may progress quickly to multidrug therapy because of continued poor seizure control. Targeted surgical excision of the epileptogenic zone in carefully selected medically refractory patients has been shown to improve outcome with 44% to 72% of patients remaining seizure free compared with 4.3% to 12% of patients on further trials of anti-epileptic medications (6, 108, 111). Despite these figures, there remains a large proportion of epileptic patients who continue to suffer unpredictable recurrent seizures. Understanding the molecular biological changes underlying the process of epileptogenesis is crucial to the development of newer, more effective treatment regimes.

## GLOBAL EXPRESSION PROFILING PLATFORMS

SAGE and DNA microarray generate large libraries of mRNA sequences enabling researchers to generate differential gene expression lists in disease states by statistical comparison of transcript frequencies between two or more conditions. Both originally described in 1995, the platforms have been extensively used in the study of human disease with millions of tags analyzed and compared with easily accessible online tag libraries. Massively parallel signature sequencing (MPSS) was developed in 2000 as an alternative quantitative open-ended platform and has risen in popularity as a research tool. A brief description of each platform follows and comparison of their relative advantages or disadvantages can be seen in Table 1.

**Table 1:** Global platforms comparison. Abbreviations: MA = microarray; MPSS = massively parallel signature sequencing; SAGE = serial analysis of gene expression.

	SAGE	MA	MPSS
Global expression profiling platforms	Yes	Yes	Yes
Prior knowledge of genome required	No	Yes	No
Specificity	80%	Near 100%	95%
	(14 nucleotide sequence)	(?Selection bias)	(17 nucleotide sequence)
Sequencing error	1%	<1%	7%
Unique transcipts/genes	Yes	No	No
Quantification	Yes	Yes	Yes
Dataset size	20–60 000 tags	Preselected tags	>1 000 000 sequences
Low abundance genes	Not sequenced	Sequenced	Sequenced
Starting mRNA concentration	High	High	DNA megacloning
	(2.5–5 μg)		
Cost	+++	+	+
Online comparative libraries	Available	Available	Available

### SAGE

SAGE as originally described is based on two basic assumptions: (i) that a short nucleotide sequence tag (9–10 base pairs) contains sufficient information to uniquely identify a given transcript; and (ii) concatenation or linking of the so-identified short sequence tags allows the efficient analysis of transcripts in a serial manner by the sequencing of multiple tags within a single clone (103).

Practically, the genome of interest is interrogated from a defined four-base pair position, classically the *NlaIII* site. An anchoring enzyme binds to this defined complementary position and acquires adjacent short mRNA sequences (9–10 base pairs) that, based on the first assumption earlier, allows specific mapping of these transcripts to genomic libraries. Proportionate amplification of different transcripts gives a realistic quantification of gene expression for the particular experiment or disease condition (67, 103).

SAGE has been utilized extensively in the study of human disease. In the first 5 years since its inception, close to five million tags had been analyzed using this method (102). It has been used in cancer, cardiovascular and immunological studies (74, 76, 77). Large online SAGE libraries are now freely available to the public, with access to raw SAGE sequence data, precomputed tag extractions and several modest analysis tools. SAGEmap currently contains over two million tags from 47 SAGE libraries and is currently available at http://www.ncbi.nlm.nih.gov/sage(56).

In terms of data analysis, SAGE is extremely efficient with a complete genome investigation allowing both whole activated and inactivated pools of gene analysis in a quantitative and qualitative manner. It does not rely on previously identified sequences or genes, thus allowing analysis and identification of novel previously unidentified genes/express sequence tags (ESTs) (20, 66, 67, 103).

Several possible key pitfalls have been identified in the SAGE technique. A high amount of starting mRNA is required that is often prohibitive and not feasible, especially in difficult to obtain human specimens or complex experimental procedures. In addition, the cost of SAGE is high and may preclude repeat experimentation to verify collected data. Although SAGE studies the entire genome, the use of a four-base pair anchoring enzyme relies on the fact that the majority of mRNA sequences contain an anchoring site on average every 256 base pair stretch (4^4^ = 256). The reality is a small proportion of mRNA will not include this anchoring site, leading to a loss of some small fraction of mRNA from the analysis that is extremely difficult to quantify. Clearly the use of a 10-base pair sequence to map gene assignment may cause issues in terms of specificity and sensitivity. It is not uncommon for multiple genes to share the same tag or for a single gene to be mapped by multiple tags.

There have been several modifications to the original SAGE technique aimed at addressing the potential pitfalls. MicroSAGE and SAGE-Lite were developed to allow lower amounts of starting mRNA to be used allow significantly smaller amounts of human or animal specimens to be examined (76, 78), whereas LongSAGE and Robust-LongSAGE were developed to improve tag or gene mapping by generating tags of 21 base pairs as opposed to 14 (35, 85, 105).

### Microarray

Microarrays are ordered samples of DNA with each sample representing a particular gene. These arrays are then assayed for changes in gene expression following experimental treatment or in different disease states. It allows simultaneous monitoring of thousands of genes, thus providing a functional aspect to sequence information in a given sample (23). There are several types of microarray technologies currently in use with studies using oligonucleotide microarray technology, the most common for high-throughput quantitative studies of RNA expression (47). In this instance, RNA extracted from the material of interest is reverse transcribed to complementary DNA and incubated with a mixture of fluorescently labeled markers at set conditions on a microarray chip containing a predetermined set of genes. Quantification is then performed by computerized measurements of fluorescent intensities.

It was first described by Schena *et al* in 1995 who purported it to be a method for quantitative measurement of expressed genes (88). Schena *et al* utilized the EST database (7) freely released from the National Center for Biotechnology Information that at the time contained a total of 322 225 entries, including 2 555 645 from the human genome. As of June 2, 2006 this count had increased to 36 750 628 total entries including 7 741 746 from the human genome and 4 719 380 from the mouse genome.

Microarray is a relatively simple procedure to perform and is becoming a standard technology for research laboratories worldwide. They allow testing of thousands of genes simultaneously with quantification of differential expressions at a reasonable cost when compared with SAGE. However, microarray technology relies on prior knowledge of the genes to be identified with the possibility of selection bias during creation of the cDNA array. As a result, novel genes are not able to be identified. Second, the amount of starting material required was also prohibitively high in the initial stages. This has largely been addressed with a two-stage hybridization process or global RNA amplification prior to running the array (73, 100).

### MPSS

MPSS was developed by Brenner *et al* in 2000 to address the variabilities, cost and scale of effort required by other high-throughput, genomic profiling methods. It utilizes the technology of DNA megacloning in combination with non-gel-based signature fluorescence sequencing to generate very large numbers of short read-length sequences (9). This process of megacloning allows the *in vitro* cloning of fragments of cDNA onto microbeads as opposed to biological hosts. Millions of cDNA fragments can be cloned by hybridization of uniquely tagged molecules to microbeads carrying 10^6^ complementary sequences to each oligonucleotide primer used (10).

MPSS has been used to create large databases of gene expression in adult human tissues (50). These have proved complementary to other database sets published using Affymetrix chip technology (89, 92, 93). It has been used to document the transcriptome in human disease states including oncology (49, 61), renal disease (30) and neuroembryology (11).

Similar to SAGE, massively parallel signature processing does not require previous knowledge of any sequence to allow analysis. The only requirement is that the microbead library is constructed large enough to contain nearly all sequences from the library to be compared against. It is time efficient with simultaneous screening of all genes and counts virtually all mRNA molecules. The libraries created are more specific as MPSS generates a 17-nucleotide signature length (95% unique) as compared with a 14-nucleotide signature length (80% unique) in a conventional SAGE. MPSS also allows the creation of an extremely large data set of signature sequences (>1 000 000) by providing a depth of analysis that allows low abundance genes to be accurately quantified, something that SAGE has been criticized for missing with datasets being comprised of only 20 000 to 60 000 sequenced mRNAs (9, 83).

Quantification of sequences is possible as MPSS generates a digital output. Differential expression comparisons can be formed with ease between samples with detection levels for statistical significance possible even with low abundance genes expressed at 30 to 40 copies per million. In contrast, hybridization techniques require replication of experiments, high abundance genes and large differences to provide adequate quantification (83).

Reproducibility of the levels of gene expression is generally very good for signatures detected in all samples. However, cases with a zero signature count have been noted to demonstrate a significantly higher error rate than those with non-zero figures. It remains unknown as to whether this represents a true measurement or the absence of measurement. In cases with conflicting values (ie, one MPSS run claiming a value and the other claiming zero), the trends obtained by ignoring the zero measurements within runs of the same sample, are shown statistically to be closer to the ideal unbiased comparisons between two samples. As such, taking the maximum number of two runs has been suggested (83, 91).

## ANIMAL MODELS OF EPILEPTOGENESIS

Seizure induction and epileptogenesis have been modeled extensively in animals. Epileptogenic substances such as alumina gel, penicillin, bicuculline, kainic acid, tetanus toxin, pentylenetetrazol and pilocarpine were directly injected to induce a primary focus of epileptogenic activity in the immediate area of placement with corresponding marked neuronal loss and gliosis, a process termed chemical kindling (60, 70). This was more often than not accompanied by behavioral seizures including status and subsequent spontaneous seizures, and is also described as the “status epilepticus models.” Other methods include the electrical kindling models first described by Goddard *et al* in 1967, whereby repeated stimulation of selected regions of the mesial temporal lobe structures result in behavioral seizures and the classical neuropathological changes seen in the human MTLE. The electrical kindling models also demonstrate seizures with progressive increase in frequency and severity following prolonged stimulation. A chronic increase in seizure susceptibility is seen; however, in contrast to the status epilepticus models, spontaneous seizures are unusual and not expected.

### Status epilepticus models

Status epilepticus models of epileptogenesis are widely used as a model of acute epileptogenesis following the application of chemical kindling. It becomes a useful model of chronic epileptogenesis with the appearance of spontaneous seizures at a time point distant to the initial convulsive event. This model demonstrates the classical progression of human temporal lobe epilepsy with an initial convulsive event leading to seizures and status epilepticus before a latency period of varying length and appearance spontaneous seizures following this. Advocates of this model point to the presence of a latency period as the period of opportunity, whereby interruption of the epileptogenic process during this time may bring about a stop in the progression of spontaneous seizures. The difficulty lies in measuring the duration of each animal's individual latency period and hence the optimal time point for intervention and study. The duration of latency is known to be related to the convulsant dose with higher doses yielding shorter onset latencies (39). Current figures suggest a mean onset latency of around 40 days; however, shorter periods have been described (32). As such, estimates of latency periods used in research studies vary from 8 to 50 days.

Mathern *et al* performed the only electrophysiological study looking into the time course of hippocampal interictal spike frequency after intrahippocampal kainite injection and correlated this with histopathological changes. Four stages were classified from this with: (i) the acute phase relating to the first 10 days after kainite-induced status epilepticus; (ii) the active phase from days 10 to 30; (iii) the latent phase relating to the latency period (days 30 to 90); and (iv) the chronic phase with the development of chronic hippocampal seizures (after 90 days). Interictal spike frequency was noted to drop during the latent phase and increase again during the chronic stage in concert with the development of behavioral seizures, neuronal loss and mossy fiber sprouting. (69)

Status epilepticus models also demonstrate a varying clinical response of the animal subjects to differing doses of convulsive agents. A high mortality rate is not unexpected and severe neuronal loss is often encountered histologically. Initial seizures are unpredictable in their duration and severity and may be difficult to control. Acute antiseizure medications such as benzodiazepines are often needed to bring about a cessation of the status epilepticus state. It is clear, however, that the onset of spontaneous seizures is directly related to the duration of the status epilepticus state and at least 30 minutes duration of initial status epilepticus is required for manifesting an epileptogenic process (53).

### Electrical kindling models

Kindling as reported by Goddard (34) refers to the repeated administration of subconvulsive electrical stimuli resulting in progressive development of seizure activity traditionally performed using daily stimulations. The term is therefore quite specific to low level electrical stimulation. Numerous alterations in stimulating paradigms have been used since Goddard's initial description of kindling and it is now well established as a chronic animal model of MTLE. It is accepted as a functional model of epilepsy where altered neuronal response develops in the absence of the gross morphological damage seen in many other epilepsy models.

The discovery of electrical kindling placed the process of epileptogenesis under greater control by the experimenter. The initial electrical kindling current stimulus was applied to a defined area and was, by definition, subconvulsive (33, 34). With repeated application of the electrical stimulus once or twice a day for only seconds at a time, electrical afterdischarges were observed to appear and gradually lengthen, and recordable after-discharges were observed to broaden in origin from adjacent brain regions to more distant regions and eventually the opposite hemisphere. In addition to this electrical progression, a behavioral progression of seizure activity occurred commencing with focal seizure activity typically manifested as initial facial clonus contralateral to the side of stimulation after a short delay. Progression through a stepwise series of more overt and eventually generalized seizures follows. This behavioral progression has been assigned a grading system, from grade 1 to 5 by Racine (81) in his study of amygdala kindling in rats. An animal may be regarded as fully kindled after manifesting a number of grade 5 seizures.

There are many advantages of the kindling model for epilepsy research. Development of chronic epileptogenesis by a precise focal activation of the target brain site is readily achieved with no mortality and very little morbidity. The pattern of seizure propagation and generalization is readily monitored and interictal, ictal and postictal periods are easily manipulated. Furthermore, the absence of the classical neuropathological changes and placement of the stimulating electrode at an area distant from the anatomical area of interest (eg, amygdala kindling for investigation of hippocampal pathology) ensures genomic expression studies are as unadulterated as possible.

Kindling experiments, however, are relatively labor-intensive, requiring a two-stage electrode implantation–electrical kindling process usually spaced over 1 to 2 weeks. Stimulation paradigms are also spaced over minutes to days and may take months to reach the fully kindled state. The ideal animal model for human temporal lobe epilepsy would consist of similar pathology, a latent period after the initial insult appropriate for the species, a state of chronic hyperexcitability and the emergence of spontaneous seizures after a latent period (107). The absence of a true latent period in electrical kindling is in opposition to this. Latency is seen from the first kindling stimulation to the eventual appearance of seizures; however, additional insults in the form of further electrical stimuli are being provided leading up to and merging with the evoked seizures. Electrical kindling also does not produce spontaneous seizures, unless prolonged kindling is performed. This is coined the “over-kindling” model (79). In this model continued electrical stimulation is given beyond the standard stage-5 criterion of the fully kindled state. The number of additional stimulations is extremely variable with recent reports suggesting at least an additional 90 to 100 kindled seizures required in rats for recurrent spontaneous seizures to occur (71, 87).

### Neuropathological changes in animal models

Similar to human changes there may be considerable variability in neuropathological changes. Status epilepticus models classically demonstrate concordant human histological changes of severe neuronal loss, granule cell dispersion, remodeling of mossy fiber structures and neurogenesis. The degree of neuronal loss is related to the concentration of inciting agent and duration of status epilepticus. Neurogenesis has been reported without corresponding cell death whereas granule cell dispersion may occur without evidence of neurogenesis (72, 98).

Electrical kindling models typically do not demonstrate evidence of neuronal loss, the advantage of which is purported of enabling focused study into the effects of seizures alone without the changes associated with cell death.

## GLOBAL GENE EXPRESSION AND EPILEPSY

An increasing number of laboratory and clinical studies involving MTLE have been published since the inception of global expression profiling platforms. Prior to this the focus of each laboratory was limited to one or a few genes selected by hypotheses generated from the available literature or laboratory experience, using conventional molecular biological techniques. These global expression studies are summarized in Table 2.

**Table 2:** Papers. Abbreviations: 2DE = two-dimensional electrophoresis; MTLE = mesial temporal lobe epilepsy; RT-PCR = real-time polymerase chain reaction; SAGE = serial analysis of gene expression.

Author	Year	Species	Model	Platform	Differential genes		Time-points
Sandberg *et al*	2000	Mouse	Pentylenetetrazole kindling	Microarray	12 or 49 out of 13 069		1 h
Liang *et al*	2001	Mouse	Rapid amygdala kindling	Differential display	26 out of 30 000 bands		0.5 h, 1 day
				RT-PCR			1 week, 1 month
Hendriksen *et al*	2001	Rat	Angular bundle kindling	SAGE	79 out of ∼6000		8 days
Potschka *et al*	2002	Rat	Amygdala kindling	MPSS	264 out of 5696		2 h
Tang *et al*	2002	Rat	Kainate kindling	Microarray	276 out of 3869		1 day
Elliott *et al*	2003	Rat	Pilocarpine Kindling	Microarray	129 out of 8000		14 days
Becker *et al*	2003	Rat	Pilocarpine Kindling	Microarray	∼400 or 700 out of 8799		3 days
					∼50 or 400 out of 8799*		14 days
Lukasiuk *et al*	2003	Rat	Amygdala kindling	Microarray	282 out of 5000		1, 4 and 14 days
Arai *et al*	2003	Rat	Ihara epileptic rat	SAGE	21 out of ∼3800		Chronic
Hunsberger *et al*	2005	Rat	Kainate kindling	Microarray	99 out of 1561		24 h
Wilson *et al*	2005	Rat	Kainate kindling	Microarray	9 out of 23 (neuropeptide probes only)		1, 6, 24, 72 and 240 h
Gorter *et al*	2006	Rat	Dentate gyrus kindling	Microarray	*CA3*	*Entorhinal*	
					2178	2548	24 h
					1400	2240	1 week
					1236	682	3–4 months
					(out of 10 179)		
Jamali *et al*	2006	Human	MTLE	Microarray	6 out of 2000		Chronic
Arion *et al*	2006	Human	MTLE	Microarray	70 out of 14 500		Chronic
Ozbas-Gerceker *et al*	2006	Human	MTLE	SAGE	146 out of ∼9500		Chronic
Greene *et al*	2007	Rat	Pilocarpine Kindling	2DE	Heat shock protein B1		2 days
Liu *et al*	2008	Rat	Pilocarpine Kindling	2DE	41 unique proteins		12, 72 h
Eun *et al*	2008	Human	MTLE	2DE	Mitochondrial SOD		Chronic

### Gene expression in epileptogenesis

#### Microarray

The predominant profiling platform utilized in global expression profiling of epilepsy and epileptogenesis has been microarray. A Pubmed search utilizing the terms “microarray” and “epilepsy” or “seizures” revealed 49 reports from which there were 10 articles publishing results of global expression profiling in models of MTLE. Bibliographic review of these articles revealed an additional two articles giving a total of 12 reports.

The majority of microarray papers published with regard to epileptogenesis involve chemical kindling. These differ in terms of model used (animal type, epileptogenic substance), time points and anatomical region investigated. Sandberg *et al* in 2000 demonstrated mouse strain-specific differences in response to pentylenetetrazole kindling; however, this study focused on the changes invoked early in the response to a single seizure and gives limited insight into the actual process of epileptogenesis.

Of more relevance are the three studies by Elliott *et al* in 2003, Becker *et al* and Tang *et al*, which in addition to characterizing genomic changes in response to chemical kindling (pilocarpine: Elliott, Becker; kainic acid: Tang), directly compared the genomic expression changes with certain physiological and disease states. In this manner, several assertions were made. Elliott *et al* suggested support for the hypotheses of parallels existing between gene expression underlying development and epileptogenic plasticity during the latency period following comparisons with the adult and developing rats (4, 24, 95).

Direct comparison of the genomic responses of brain in response to ischemic stroke, intracerebral hemorrhage, kainic acid induced seizures, hypoglycemia and hypoxia was made by Tang *et al* in 2002. Significantly, marked overlap of regulated genes was found throughout all disease states. In particular, all genes induced by kainic acid were also induced by ischaemia, hemorrhage or hypoglycemia. This suggests a multifactorial mechanism of injury for kainic acid induced seizures that mimics or utilizes the same pathways as those associated with ischaemia, hypoglycemia or hemorrhage (95). Whether seizures themselves primarily induce these genomic changes or secondarily act through periods of ischemic or hypoglycemic during seizure episodes remain unclear. What is clear, however, is the similarities in histological changes seen in these conditions with marked neuronal loss, fibrillary gliosis with vacuolation and enlarged reactive astrocytes variously reported following severe hypoglycemia and ischemia, with or without associated status epilepticus (1, 13, 51, 52).

Becker *et al* correlated temporal changes of genes associated with cellular stress and injury at 3-day post-status epilepticus, genes associated with cytoskeletal and synaptic reorganization at 14-day post-status epilepticus, and genes involved in neurotransmission pathways at the chronic epilepsy stage. This study also provided the first comparison with human hippocampal specimens with eighteen genes differentially expressed in both the chronic stage of pilocarpine induced epilepsy and medically refractory MTLE (4).

Other experimental microarray studies investigated changes of growth factor signaling genes, transcription factors, angiogenesis signaling molecules, neuropeptides, neuronal plasticity and signal transduction using custom-made probe sets at varying time points. These are summarized in Table 2(44, 63, 110).

The electrical kindling model was utilized by Lukasiuk *et al* to investigate the gene expression changes during specific phases of epileptogenesis with the hypothesis that remodeling of neuronal circuits underlying epilepsy is associated with altered gene expression during epileptogenesis. RNA was extracted from the hippocampus and temporal lobe at 1, 4 and 14 days following electrical kindling and hybridized with cDNA arrays containing approximately 5000 rat gene probes. There were 87 differentially expressed genes within the hippocampus with 37 genes regulated at 1 day, 12 at 4 days and 14 at 14 days. There was an overlap of 13 genes with the temporal lobe that demonstrated 208 differentially expressed genes in a similar time distribution as the hippocampus genes. Functional annotation of the regulated genes revealed genes involved in neuronal plasticity, gliosis, organization of the cytoskeleton or extracellular matrix, cell adhesion, signal transduction, regulation of cell cycle and metabolism (63).

#### SAGE

A literature search using the words “serial analysis of gene expression” and “epilepsy” and/or “seizures” produced four articles. Two articles were published from the same laboratory with one expanding on the initial report, whereas the other two studies involved human MTLE and the use of a genetically modified epileptic rat, the Ihara rat.

There is only one published SAGE report using the rat electrical kindling model of mesial temporal lobe. Unlike most electrical kindling models, marked neuronal cell loss and gliosis is seen histologically in the electrical kindling model utilized by Hendriksen *et al* with a latent period of between 1 and 2 weeks following electrical kindling of the angular bundle and the appearance of spontaneous seizures. As such, hippocampal harvest was performed 8 days after the final electrical stimulation, prior to the commencement of spontaneous seizures and during the process of epileptogenesis. Over 10 000 tags were analyzed in both stimulated and control groups resulting in SAGE libraries containing 5053 and 5919 different unique tags respectively. There were 92 differentially expressed genes, predominantly associated with ribosomal proteins, protein processing and axonal growth and glial proliferation (42).

SAGE was also performed on hippocampi of the Ihara epileptic rat (IER). The IER is an animal model of MTLE that demonstrates progressive limbic seizures, eventually resulting in spontaneous generalized tonic-clonic seizures at 5 to 6 months of age. SAGE was performed on hippocampal tissue taken at 2 months of age and compared with the Wister rat. Over 7000 tags were analyzed with creation of a SAGE library for each group consisting of 2492 and 2141 genes, respectively. Eighty-one differentially expressed genes were identified, with genes associated with neurotransmission and intercellular component downregulated, and genes associated with protein synthesis, metabolism, membrane transport, the cytoskeleton and ion-channels upregulated (2).

Our laboratory has performed SAGE on adult male C57/BL6 mice following rapid electrical kindling of the amygdala. This model of MTLE is useful in allowing assessment of the effect of seizures during epileptogenesis without the confounding effects of neuronal loss and cell death (90). RNA was extracted from hippocampi taken 3 h following the completion of kindling with around 28 000 tags analyzed in control and stimulated groups. In the control group, 13 213 unique tags were identified whereas 12 302 unique tags were identified in the stimulated group. There were 55 differentially expressed genes when comparing between the groups with functional classification denoting immune response, cellular metabolism, axonal growth and regeneration, signal transduction, ion transport, synaptic and neurotransmission and genes of unknown identity or function predominant (Wang *et al*, in preparation).

#### MPSS

There has been a single published report using MPSS for gene expression studies during epileptogenesis. Potschka *et al* utilized the rat kindling model of epileptogenesis to characterize the genomic changes using MPSS. Seven thousand and four signatures with a minimal occurrence of two clones in each run were analyzed resulting in annotation of 5696 identified genes. Two hundred sixty-four differentially expressed genes were identified with 128 upregulated and 136 downregulated ones. The immediate early gene Homer 1A was most strongly induced at 2 h. Identification of this gene fueled further research into the possibilities of Homer 1A being an intrinsic anti-epileptogenic and anticonvulsant gene upregulated as a protective measure during epileptogenesis (80).

## HUMAN STUDIES

There remains a relative dearth of global gene expression studies involving human specimens. This is somewhat surprising given that mesial temporal lobectomy, hippocampectomy and amygdalectomy is a well-accepted treatment option for medically refractory MTLE in carefully selected cases giving a readily available source of hippocampal specimens (96). Four studies have been reported utilizing microarray (3) and SAGE (1) of which only three dealt with MTLE patients.

Jamali *et al* analyzed the entorhinal cortex from the hippocampus of brains removed as a surgical treatment consisting of a standard anterior temporal lobectomy whereas Özbas-GerÇeker *et al* did not differentiate between hippocampal subregions. Both studies attempted to find control tissue in the form of autopsy hippocampal specimens; however, this obviously posed a significant problem in terms of reproducibility of “controls” not to mention the ethical dilemma. In an attempt to factor in a bias related to long-term exposure to anti-epileptic medications, Jamali *et al* also compared samples from autologous adjacent lateral temporal cortex that had been subjected to the same environment as the epileptic tissue.

Comparison of the entorhinal cortex with non-epileptic lateral temporal lobe revealed 16 regulated genes of which 10 were upregulated and six were downregulated. This study suggested dysregulation of the neurotransmission and complement systems within the entorhinal cortex as another pathway affected in the process of epileptogenesis (46). On the other hand, the study by Özbas-GerÇeker *et al* revealed 143 differentially expressed genes that matched functionally to genes associated with basic metabolism, transcription regulation, protein synthesis and degradation, signal transduction, structural proteins, regeneration and synaptic plasticity and genes of unknown identity or function (74).

Neither study attempted to perform a comparative approach using animal models with their human tissue that would add validity to any prior or future reports into this topic. On the other hand, the study by Becker *et al* in 2003 demonstrated complimentary results between rat and human specimens with 18 genes regulated in the chronic stage of the rat status epilepticus model showing corresponding expression patterns in hippocampal subfields of patients with pharmacoresistant MTLE (4).

## PROTEOMIC STUDIES

Proteomics is a technique that enables one to find proteins changed by the cells response to internal states, external stimulations or developmental cortex. The technique of the two-dimensional electrophoresis (2DE) allows global profiling of the proteomic changes in different disease states or conditions. Two studies into chemically induced epilepsy (pilocarpine) have investigated the proteomic profile following epileptogenesis in the whole hippocampi alone or in conjunction with the forebrain in rats. These suggested upregulation of pathogenic, neuroprotective and neurogenic responses with validation performed using Western blotting and immunohistochemistry (36, 59).

A single study by Eun *et al*(25) attempted to analyze human temporal lobe specimens in patients with MTLE undergoing surgical resection by performing 2DE and Western blotting of the selected proteins. Control specimens were obtained from lateral temporal cortices of patients undergoing temporal lobe operations for tumors in the absence of seizures. This study reported significant changes in nine proteins with particular emphasis on proteins related to oxidative stresses; it also highlights the shortcomings in performing comparative studies in humans with control specimens coming from a variety of different diseases such as glioblastoma multiforme (7), sphenoid wing meningiomas (3) and malignant lymphomas (2).

## SUPPORTIVE OR CONFLICTIVE

**Figure 3:**
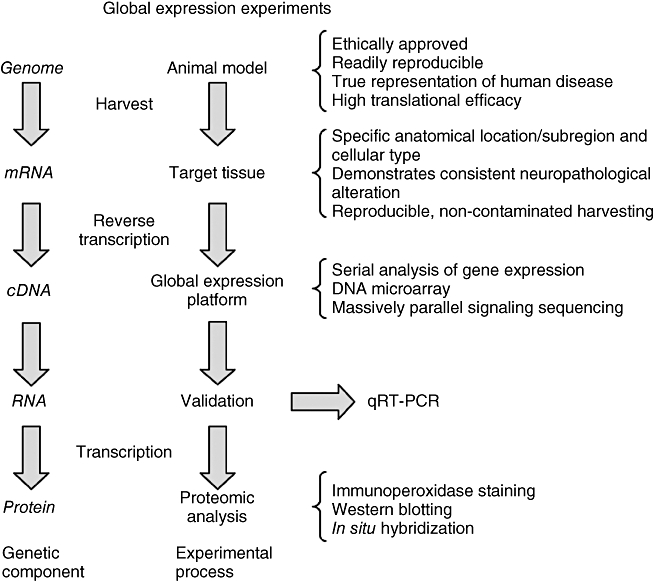
*Global expression profiling experiments.* Abbreviations: qRT-PCR = quantitative real-time polymerase chain reaction.

The advent of global expression profiling technology has led to an ever increasing amount of genomic information available. To the end of 2006, there are over 1100 published reports on microarray studies involving the brain and brain disorders, with nearly 100 SAGE studies of the same. For epilepsy studies the figures reveal 32 microarray and four SAGE studies (Figure 3).

Yet for all the additional knowledge that has been acquired, the outcomes of epilepsy treatment, particularly MTLE, remain in the main stagnant. There is as yet no active intervention to prevent progression of epilepsy either from the “initial insult” to the “latency period,” or from the latency period to chronic epilepsy. The question follows then: what is the benefit of large-scale gene expression profiling? Is the accumulation of knowledge of benefit to the treating epileptologist or does it create more confusion in the treatment of this already complex condition?

The inherent problems with analyzing and applying data generated from global expression profiling stems from the obvious difference in epilepsy models, the profiling platform used, animal type and laboratory environment. Care must be taken in comparing genomic profiles generated from differing epilepsy models. Genomic reactions are clearly different between different species of the same animal (86). These differences must be accentuated even more when comparing the different profiles in mice, rats and humans. Status epilepticus models of epileptogenesis possess the advantage of a clear-cut progression from initial insult to chronic epilepsy in contrast to electrical kindling where a predominantly time-dependent protocol is usually administered. The rapid electrical amygdala kindling model utilized in our laboratory allowed the effects of seizures in isolation to be investigated without the confounding effects of neuronal loss. In contrast, status epilepticus models of epileptogenesis demonstrate marked neuropathological changes and may manifest widespread systemic effects. The use of benzodiazepines to halt prolonged severe status epilepticus in these models may also adversely alter the transcriptome.

Tang's comparisons between kainic acid-induced seizures, hypoxia, hypoglycemia and hemorrhage suggest that the kainic acid-induced changes utilize the same pathological pathways as the other three conditions (95). Indeed there are clear interrelations between all such conditions with prolonged seizures resulting in metabolic acidosis and an environment of relative hypoxia and hypoglycemia, and prolonged and severe hypoglycemia resulting in seizures in the generalized tonic-clonic form. The eventual histopathological outcome from these events culminates in varying degrees of neuronal loss, gliosis and even necrosis. If this is the case, it is difficult to proclaim that the genetic expression changes are actually specific to the process of epileptogenesis. Another study reported in abstract form (21) and cited in Lukasiuk *et al*'s review revealed distinct differences in gene expression between the kainic acid and pilocarpine models of epileptogenesis, despite being studied in as exacting an environment of the same laboratory.

The pathology of HS deserves special mention at this point. It remains a point of controversy as to whether HS is the underlying cause, or end result of the clinical syndrome of MTLE. As a cause, the proposed theories underlying HS include childhood febrile convulsions, perinatal hypoxic events or vascular accidents, whereas as an end result it is suggested repeated seizures progressively inflict end-organ damage, particularly in the susceptible areas of the hippocampus described earlier. In a molecular biological sense, a causative action would allow quantification of gene expression changes of each cell type whereas as an outcome, genomic profiling would be representative of changes in cell population.

Confounding the arguments are the documented cases of normal hippocampal size and histology on positively electrophysiologically localized epileptogenic foci excised as a curative procedure (15) as well as autopsy specimens demonstrating histological changes consistent with HS in the absence of clinical seizures (48). The unique cerebral responses in different individuals must be kept in mind in all laboratory and translational research studies.

The number of published differentially expressed genes during epileptogenesis is fast approaching 2000. On review of the available data from these publications there is minimal conformity between the genes described. Lukasiuk *et al* has produced a list of common differentially expressed genes involving models of epileptogenesis, including traumatic brain injury studies (62). From the near 2000 genes, only a small number (53) are noted to be differentially expressed in more than one study. However, if the gene profiles of traumatic brain injury studies are filtered out, only 38 genes remain differentially expressed across studies. In addition, a proportion of these common genes appear to be differentially expressed in conflicting directions.

In order to further assess the consistency of altered gene expression across the studies we annotated the list of commonality differentially expressed genes to include studies published to the end of 2006 as well as our currently unpublished SAGE data. Comparisons were performed using all available published lists as well as accessing complete gene lists made available through online downloads. Differences in epilepsy models and global expression profiling platforms were also recorded. There were 72 genes reported to be regulated during epileptogenesis in two or more studies. Thirty-nine genes demonstrated a consistent direction of change with 15 showing conflicting results. The direction of regulation could not be ascertained from the available literature in 20 genes. No genes were reported regulated in more than five papers with the majority (50) only regulated in two papers. The two genes consistently regulated in five reports were the rather ubiquitous glial fibrillary acidic protein and the S100-related protein (Table 3).

**Table 3:** Genes in more than one study. Abbreviations: IER = Ihara epileptic rat; MA = microarray; SAGE = serial analysis of gene expression.

Gene name	Abbreviation	Reports	Platform		Conflicting	Epilepsy model			
			SAGE	MA		Electrical kindling	Chemical kindling	Human	IER
*14-3-3 protein gamma*		2	1	1	No	1	1	0	0
*Actin related protein 2/3 complex*	*Arpc*	3	1	2	No	1	2	0	0
*α*-*tubulin*		2	1	1	No	1	1	0	0
*Brain derived neurotrophic factor*	*BDNF*	4	1	3	No	2	2	0	0
*Calponin 3, acidic*	*Cnn3*	2	1	1	No	1	1	0	0
*Cathepsin D*	*Ctsd*	2	1	1	No	1	1	0	0
*Cathepsin S*	*Ctss*	2	1	1	No	1	1	0	0
*Cholecystokinin*	*CCK*	3	1	2	No	1	0	2	0
*Chromogranin B*	*Chgb*	2	1	1	No	1	1	0	0
*Clone BB.1.4.1*		2	1	1	No	1	1	0	0
*Complexin 2*	*Cplx2*	2	2	0	No	1	0	1	0
*Cystatin C*	*Cst3*	4	2	2	No	2	2	0	0
*Ectodermal neural cortex*	*Enc1*	2	1	1	No	0	0	2	0
*Elongation factor-1α*	*Efa1*	2	1	1	No	1	1	0	0
*Epithelin 1 and 2*		2	1	1	No	1	1	0	0
*ESTs, Hs to MBD3*		2	1	1	No	1	1	0	0
*Ferritin heavy chain*	*Fth*	3	2	1	No	2	1	0	0
*Glial fibrillary acidic protein*	*GFAP*	5	2	3	No	1	3	1	0
*Granulin*	*Grn*	2	1	1	No	1	1	0	0
*Growth factor receptor bound protein 2*	*Grb2*	2	0	2	No	0	1	1	0
*Heat shock protein 27*	*Hsp27*	2	0	2	No	0	2	0	0
*Heat shock protein 70*	*Hsp70*	4	1	3	No	1	2	1	0
*Metallothionein 2*	*Mt2*	3	0	3	No	0	3	0	0
*Metallothionein 3*	*Mt3*	2	2	0	No	1	0	1	0
*MHC class Ib antigen*		2	0	2	No	0	2	0	0
*Myelin basic protein*	*Mbp*	3	2	1	No	1	1	1	0
*NDRG family member 2*	*NDRG2*	2	1	1	No	0	0	2	0
*Neural precursor expressed, developmentally downregulated gene*	*Nedd*	2	2	0	No	1	0	1	0
*p41-Arc*	*arpD*	2	0	2	No	0	2	0	0
*Pleckstrin*	*Pscd1*	3	2	1	No	1	0	2	0
*Preprocathepsin*		2	1	1	No	1	1	0	0
*Protein p9Ka homologous to calcium-binding protein (S100)*	*S100*	5	1	4	No	2	3	0	0
*Secreted protein, acidic, cysteine-rich (osteonectin)*	*SPARC*	4	2	2	No	1	2	1	0
*Spinophilin*	*Spn*	2	1	1	No	1	1	0	0
*Tachykinin 1*	*Tac1*	2	0	2	No	0	2	0	0
*Thyrotropin releasing hormone*	*TRH*	4	0	4	No	1	3	0	0
*Tissue inhibitor of metalloproteinase 1*	*Timp1*	3	0	3	No	1	2	0	0
*Transferrin*	*Tsf*	2	0	2	No	0	1	1	0
*Vimentin*	*Vim*	2	0	2	No	0	2	0	0
*Apolipoprotein*	*ApoE*	4	3	1	Yes	3	0	1	0
*Ca(2+)/calmodulin-dependent protein kinase II*	*CaMKII*	4	1	3	Yes	2	1	1	0
*Cabonic anhydrase*	*CA*	2	1	1	Yes	0	0	2	0
*CD 99 antigen*	*CD99*	2	1	1	Yes	0	0	1	1
*Glyceraldehyde phosphate dehydrogenaase*	*GAPDH*	3	2	1	Yes	2	1	0	0
*Glycoprotein 65*		2	1	1	Yes	1	1	0	0
*Growth arrest and DNA damage-inducible protein 45*	*GADD45*	3	0	3	Yes	0	3	0	0
*Laminin receptor 1*	*Lamr1*	2	1	1	Yes	0	1	1	0
*Metallothionein 1*	*Mt1*	3	1	2	Yes	1	2		0
*Ornithine decarboxylase*	*Oda*	2	1	1	Yes	0	1	1	0
*Peptidylprolyl isomerase B (cyclophilin B)*	*Ppib*	2	0	2	Yes	0	2	0	0
*Platelet activation factor acetylhydrolase*	*Pafah*	3	1	2	Yes	0	2	1	0
*Protein kinase C β*	*PrkCB*	2	0	2	Yes	0	2		0
*Proteolipid protein 1*	*Plp*	2	1	1	Yes	1	0	1	0
*Thymosin beta*	*Tmbx*	4	3	1	Yes	2	1	1	0
*Serum/glucocorticoid regulated kinase*	*Sgk*	2	0	2	Unknown	1	1	0	0
*CD 14 antigen*	*CD14*	2	0	2	Unknown	1	1	0	0
*CD 74 antigen*	*CD74*	2	0	2	Unknown	1	0	1	0
*Corticotropin releasing hormone*	*CRH*	2	0	2	Unknown	1	1	0	0
*GABA(A) receptor associated protein*	*GABARAP*	2	1	1	Unknown	1	0	0	1
*Galanin*	*Gal*	3	0	3	Unknown	1	2	0	0
*Glutamate receptor, ionotropic, N-methyl D-aspartate 2A*	*Grin2A*	2	0	2	Unknown	1	0	1	0
*Homer homolog 1*	*Homer*	3	DD, MPSS	1	Unknown	3	0	0	0
*Neuritin*	*Nrn*	2	0	2	Unknown	0	2	0	0
*Neuropeptide Y*	*Npy*	4	0	4	Unknown	1	3	0	0
*Nuclear factor of kappa light chain gene enhancer in B-cells inhibitor, alpha*	*Nfkbia*	2	0	2	Unknown	1	1	0	0
*Peptidylglycin-alpha-amidating monooxygenase*	*PAM*	2	0	2	Unknown	0	2	0	0
*Prostaglandin-endoperoxide synthase 2*	*Ptgs2*	2	0	2	Unknown	1	1	0	0
*Secreted phosphoprotein 1*	*Spp1*	2	0	2	Unknown	1	1	0	0
*Signal transducer and activator of transcription 3*	*Stat3*	2	0	2	Unknown	1	1	0	0
*Somatostatin*	*Sst*	2	0	2	Unknown	1	1	0	0
*Synaptophysin*	*Syp*	2	0	2	Unknown	2	0	0	0
*Syndecan 4*	*Sdc4*	2	0	2	Unknown	1	1	0	0
*VGF nerve growth factor inducible*	*Vgf*	3	0	3	Unknown	1	2	0	0
*β2-microglobulin*	*β2m*	2	0	2	Unknown	2	0	0	0

The functional classification of these genes is also interesting to review. The gene ontology classification of cellular component, biological process and molecular function has been used for functional classification by the majority of authors whereas several have utilized functional groups described previously in the literature including immediate early genes/transcription factors, calcium homeostasis, intra/extracellular signaling, synaptic/vesicular, morphology, cell cycle/fate, injury/survival, metabolism and unknown. A list of the most prominent biological process regulated as described by each author is seen in Table 4. It is clear that there is a wide range of biological processes involved in the epileptogenic process. These include basic cellular metabolism, regulation of transcription, protein processing, synaptic transmission, response to injury/cell death and regeneration and immune response.

**Table 4:** Biological processes. Abbreviations: EC = endothelial cell; ECM = extracellular matrix; PG = prostaglandin.

Biological process	Author									
	Arai	Elliott	Gorter	Hendriksen	Hunsberger	Jamali	Liang	Lukasiuk	Tang	Wang
Axonal growth and proliferation				✓						
Cell adhesion								✓		
Cell cycle progression					✓					
Cell damage, axonal growth and regeneration									✓	
Cell death					✓					
Cellular metabolism	✓	✓		✓					✓	
Cellular transport										✓
Coagulation pathway			✓							
Cytoskeletal/ECM organization								✓		
EC signaling		✓								
ECM remodeling					✓				✓	
Gliosis								✓		
Immune response			✓			✓			✓	✓
Injury response/cell survival		✓								
Membrane transport	✓									
Morphology		✓								
Neuronal plasticity								✓		
Neurotransmission						✓				
PG synthesis			✓							
Protein processing				✓						✓
Signal transduction							✓			
Synaptic transmission			✓							✓
Transcriptional regulation					✓		✓			

The different time frames of the analysis of the gene expression also contribute to the differences to the profiling results. Transcriptional changes immediately following seizures will encompass the response to hyperthermia, mild hypoxia or hypoglycemia, and even direct trauma from any cerebral or truncal impacts. This contrasts with gene expression during the latent phase of epileptogenesis whereby a period of recovery with substrate replenishment and remodeling occurs, and during the chronic epileptic states seen in human studies. Numerous studies have demonstrated the transient nature of transcriptional changes within their own epileptogenesis model, let alone between different models (57, 63, 110).

Lukasiuk *et al* have recently presented in abstract form and in a review article, an extension to their previous analysis of gene expression during epileptogenesis. Identification of highly represented functional classification as well as individual genes that appeared across data sets was performed with cell death and survival, neuronal plasticity and immune response as the most prominent. Seventy individual genes were identified to be consistently regulated across at least three studies using specific time-point analyses (64, 65). Our data indicates only 22 genes consistently regulated across at least three studies. This difference is explained by the inclusion of animal models of traumatic head injuries in the analysis by Lukasiuk *et al* that we did not include. The justification for their inclusion is the initial hit theory whereby an early insult, be it ischemic, traumatic or infective, sets into motion a series of molecular changes that lead into the latency period before chronic epilepsy. However, recent figures suggest only 2% to 5% of traumatic brain injury cases will develop epilepsy (75). Second, post-traumatic seizures are more commonly a result of cerebral contusions leading to scarring and altered neuronal circuitry rather than the classical MTLE syndrome. Filtering out the head injury papers from Lukasiuk *et al*'s study leaves only four consistently regulated genes, three of which are included in our list of regulated genes (*Gadd45a*, *Ctsd*, *Ctss*, *Zfp36*). The last gene, *zinc finger protein 36* (*zfp36*) is only reported differentially expressed in different arms of the same study by Becker *et al* and was excluded from our analysis.

The complexity of neuronal structure and organization within the mesial temporal lobe regions may contribute to this issue. In particular, the anatomical subregions of the hippocampus demonstrate both histological and functional differences in their composition and response to certain conditions. Whereas CA1 and CA3 are considered integral to the transmission of input signals, CA2 is considered a transitional zone with no clear function and the dentate gyrus is considered the main input pathway into the hippocampus, receiving afferents itself from the entorhinal cortex via the perforant pathway (31). Increasing interest in gene expression profiling between hippocampal subfields has suggested a molecular basis underlying the previously defined anatomic subregions of the hippocampus (112). This may further improve understanding of the differing histopathological responses described earlier.

Few reports have attempted to stratify global gene expression profiles in terms of hippocampal subfields. Indeed, of the reviewed articles, only three reported comparisons between subregions of the brain. Reasons for this may include the large amounts of starting mRNA needed for global gene expression platforms in general, or the difficulty in isolating individual subfields. The use of stereo dissection or laser microdissection to accurately isolate different hippocampal subregions as performed by Becker *et al*, Datson *et al* and Torres-Munoz *et al* have challenged these notions. This technique has been described to allow direct microscopic identification of specific cell types without appreciable loss of their DNA or RNA. Furthermore microarray analysis was able to be performed on the limited amount of mRNA extracted using double round amplification to provide sufficient material (4, 17, 99). Marked differences (>700 genes) were found in the hippocampal response to chronic glucocorticoid exposure between CA3 and the dentate gyrus; however, 79 genes reliably identified in individual subfields were virtually undetectable when looking at whole hippocampal specimens. The practice of analyzing gross or pooled hippocampal specimens fails to take into account these obvious differences and may skew ensuing results.

Although global expression profiling has the advantages of being time efficient in analyzing large portions of the transcriptome, there are limitations to the sensitivity and accuracy. It is well documented that SAGE may inconsistently detect low abundance transcripts leaving small genomic expression changes induced by the experimental model or disease undetected. This is evident in examining the epilepsy studies discussed above with each generated database containing 5000 to 14 000 unique tags only, a significant decrease from the estimated size of the entire hippocampal transcriptome being over 30 000 (16, 26). Furthermore, the sequencing error of SAGE is estimated at an average of 1% per base because of the fact they utilize a single-pass sequence in data processing. This accumulates to roughly 10% per 100 base pairs, leading to lower correct tags counts and artificially inflate the tag counts of already established tags or establish a count of a tag that does not exist. Microarray studies have also been shown to demonstrate significant variations even under the same experimental conditions. As such, validation of global expression profiling results by independent methods, such as real-time polymerase chain reaction (PCR), Northern/Western blotting and immunohistochemical methods, is crucial. In our experience, a significant decrease in the number of differentially expressed genes is demonstrated when validating SAGE results using quantitative real-time polymerase chain reaction (qRT-PCR) with a custom-made, low-density array chip of 94 significant genes as indicated by SAGE (82).

Following the changes into the proteomic stage will further validate results of global expression profiling. Translation into functional proteins represents the final pathway of genomic regulation. Quantifying the proteomic changes specimens through immunoperoxidase stainings and *in situ* hybridizations will not only complete the interrogation process, but also give insights into region-specific changes that may have been missed in interrogation of gross hippocampal, possibly negating the requirement for microdissection of hippocampal subregions (Figure 3).

In terms of gene expression platforms, the laboratory conditions play a vital role in the reproducibility of results. It is not unusual for the same experiments performed on different days to produce differing results. Similarly, experiments performed by separate scientists on the same days will also often produce differing results. Subtle variations in the complementary tags used to interrogate the transcriptome can also cause biased results. Specifically, selection of cDNA probe sets on microarray cards or the use of modifications of the SAGE technique such as LongSAGE adds more potential for a varied result.

What then are the advantages of global expression profiling? The advent of these technologies in the mid-1990s heralded a new excitement into the possibilities of molecular biological research. It allowed researchers to access the diverse levels of biological processes in its entirety, from primary DNA sequences in coding and regulatory regions, to RNA expression in response to the physiological or diseased environment, to proteomic interactions and localizations. The sheer power of simultaneously interrogating thousands of genes enables the acquisition of global pictures of biological processes that would otherwise be out of reach utilizing traditional gene-to-gene approaches.

Prior to global expression profiling, laboratory research was being driven by theories and hypotheses developed from meticulously analyzed pathways or the occasional spontaneous brain wave. The formulation of these theories in the history of epilepsy research have resulted in much interest in the neural imbalance of excitatory/inhibitory control, or the involvement of ionic channels and aberrant neuronal synapses in the epileptogenic processes (97). Indeed, numerous laboratories have published insightful papers documenting a net excitatory imbalance in MTLE models (14, 22, 84). More recently two microarray studies revealed confirmatory findings with the downregulation of gamma-aminobutyric acid-associated receptors and the upregulation of glutamate-associated receptors in the kainite model of epileptogenesis as well as chronic human MTLE (3, 44). What is not evident in the literature are the situations where there have been failed hypotheses resulting in a laboratory and scientific losses in terms of time, personnel and finance. The use of global profiling platforms to guide hypotheses may be a viable option to minimize these situations.

The advantage in global expression profiling is the development of a broad understanding of the mechanisms underlying the process. It is the accumulation of the supportive data gathered from functional classes that enables more robust hypotheses to be formed and tested. In turn this allows further focussed research into specific genes or functional classes as has already been performed by several laboratories (42, 80). Novel treatment strategies may be identified from the identification of the involvement of functional groups with development of new pharmacological agents targeting the specific biological process.

The publication of comparable gene libraries produced using global expression profiling methods also allows indirect interrogation between epilepsy and other disorders. Identification of commonality genes across disorders may provide a greater understanding of the aetiological or consequential effect of a regulated gene/process. Detection of common pathways through which each disorder is propagated may allow cross-over treatment between disorders giving new insight and treatment options.

## CONCLUSIONS

Global expression profiling during epileptogenesis allows a greater understanding of the broad mechanisms underlying the epileptic process. It demonstrates specific genomic changes operating in concert with the entire molecular environment, thereby opening the doors to further research into possible causative pathways and functions through gene ontology annotations of cellular components, biological processes and molecular functions. In addition, the availability of large-scale libraries produced using uniform protocols allows the comparisons of commonality genes within other disorders apart from epilepsy potentially identifying reactive general genomic changes, shared pathways of action or specific causative genes. There is also the potential for discovery of novel genes not previously described with SAGE resulting in new hypotheses and theories into the pathogenesis of epilepsy as well as the potential to guide new therapeutic treatments in epilepsy as well as other disorders, neural or non-neural.

However, the most exciting aspect of global gene expression profiling is the general starting point of the research study. Previously, investigations into specific genes were performed based on previous studies or individual hypotheses. The use of global expression profiling allows the entire genome to be studied with hypotheses developed following this. In this manner, future research hypotheses are generated from science rather than hypotheses leading to science, a situation that must lend itself to greater transparency in laboratory research.

It is vitally important that further research into epileptogenesis builds on prior knowledge and pitfalls. It is the opinion of our laboratory that the entire process of molecular transcription and translation be interrogated with validation of global expression profiling results utilizing qRT-PCR followed by proteomic profiling using any combination of Western blotting, immunoperoxidase staining and *in situ* hybridization. Focusing genomic profiles into specific anatomical regions may enable causative molecular pathways to be identified and allow greater understanding of the histological changes, particular in the situation of HS associated MTLE whereby disagreement remains over the cause or effect of the pathological changes.
